# Serological Surveillance of Hospitalized Patients for Lyme Borreliosis in Ukraine

**DOI:** 10.1089/vbz.2020.2715

**Published:** 2021-03-25

**Authors:** Mariia Shkilna, Mykhailo Andreychyn, Mykhailo Korda, Olena Pokryshko, Roksolana Humenna, Mariana Huk, Shuling Liu, Artem S. Rogovskyy

**Affiliations:** ^1^ Department of Infectious Diseases and Epidemiology, Dermatology and Venereology, Virology and Immunology, I. Horbachevsky Ternopil National Medical University, Ternopil, Ukraine.; ^2^ Department of Medical Biochemistry, Virology and Immunology, I. Horbachevsky Ternopil National Medical University, Ternopil, Ukraine.; ^3^ Department of Microbiology, Virology and Immunology, I. Horbachevsky Ternopil National Medical University, Ternopil, Ukraine.; ^4^ Statistical Collaboration Center, Department of Statistics, College of Science, Texas A&M University, College Station, Texas, USA.; ^5^ Department of Veterinary Pathobiology, College of Veterinary Medicine and Biomedical Sciences, Texas A&M University, College Station, Texas, USA.

**Keywords:** *Borrelia burgdorferi*, serological assay, Ukraine, ELISA, Western blot, survey

## Abstract

***Objectives:*** Although in Ukraine the incidence of Lyme borreliosis (LB) has been surging up over the past decades, seroepidemiologic data are not available to date. The objective of this report was to perform preliminary serological survey of hospitalized population for LB.

***Methods:*** Sera were collected from 203 patients of a hospital located in Western Ukraine. Most patients showed clinical signs that were compatible with LB such as arthritis (*n* = 29), neurological signs (*n* = 35), and erythema migrans (EM)-like lesions (*n* = 60) or unrelated to LB (*n* = 79). The specimens were tested by enzyme-linked immunosorbent assay and Western blot for anti-*Borrelia* antibodies.

***Results:*** LB was confirmed in 8.6%, 34.5%, and 50% of the patients, who exhibited neurological signs, arthritis, or EM-like lesions, respectively. Anti-*Borrelia* antibodies were also detected in 6.3% of the patients with pulmonary tuberculosis.

***Conclusions:*** This study provides the first preliminary data on the seroprevalence of LB in Ukraine. Future studies are warranted to investigate more subsets of the Ukrainian population for this emerging tick-borne disease.

Lyme borreliosis (LB) is the leading tick-borne disease in the Northern Hemisphere. LB is mainly caused by *Borrelia afzelii*, *Borrelia burgdorferi* sensu stricto (*B. burgdorferi*), *Borrelia garinii*, or *Borrelia spielmanii* (Kuehn 2013, Sykes and Makiello 2017, Littman et al. 2018). The active surveillance consistently demonstrates that LB has been on the rise in Europe. In Western European countries, the average annual incidence can be as high as 22 persons per 100,000 population (Vandekerckhove et al. 2019). However, published studies on LB incidence in Eastern European countries are often absent. As an example, in Ukraine, no seroepidemiologic studies have been performed to date despite the annual LB incidence has been steeply increasing (Rogovskyy et al. 2020). This study's objective was to conduct serological survey of human patients in a hospital setting in Ukraine.

Sera were collected over 2015–2018 from 203 patients, who were admitted to a general hospital located in Ternopil (Western Ukraine). The study was approved by the bioethics committee of I. Horbachevsky Ternopil National Medical University (Ukraine). Patient consents were not required as the sera were collected as part of routine diagnostic procedure. Most patients exhibited clinical signs, which were consistent with LB such as arthritis (17 females and 12 males; the median age is 43 years), neurological signs (21 females and 14 males; 37 years), or erythema migrans (EM)-like lesions (37 females and 23 males; 42 years) (Supplementary Tables S1–S4). In addition, the study included 79 patients with pulmonary tuberculosis (47 females and 32 males; 44 years).

Anti-*Borrelia* enzyme-linked immunosorbent assay (ELISA) and anti-*Borrelia* plus VlsE ELISA test systems (Euroimmun, Germany) were, respectively, used to detect IgM and IgG antibodies against *B. afzelii*, *B. burgdorferi*, and *B. garinii*. Test results were interpreted per manufacturer's instructions. Positive or borderline samples were tested by Western blot (WB) to confirm the presence of IgM antibodies specific to *B. afzelii*, *B. burgdorferi*, *B. garinii*, and *B. spielmanii* (anti-*Borrelia* EUROLINE-RN-AT-adv, Euroimmun, Germany). Also, the ELISA-negative samples from the patients that showed arthritis or EM-like lesions and that had fever or fatigue, or were bitten by a tick, were also tested by WB.

For the patients, who were tested by IgM ELISA, IgG ELISA, and WB (the patients with arthritis or EM-like lesions), four mutually exclusive groups were identified: tested positive by WB regardless of IgG or IgM ELISA results; tested positive by IgM or IgG ELISA but negative by WB; tested negative by IgM ELISA, IgG ELISA and WB and others (Table 1). Age, gender, duration of the symptoms, and history of tick bites were analyzed using descriptive statistics. Differences for continuous and categorical variables were tested through ANOVA and Fisher's exact test, respectively (SAS version 9.4; SAS Institute, USA). We also performed pairwise comparison for selected pairs of the four groups. In particular, we compared the following pairs: (1) patients who tested positive by WB only vs. those who tested negative by WB but positive by IgM ELISA and/or IgG ELISA; (2) patients who tested positive by WB only vs. those who tested negative by WB, IgM ELISA and IgG ELISA; (3) patients who tested positive by IgM ELISA or IgG ELISA but negative by WB vs. those who tested negative by WB, IgM ELISA and IgG ELISA.

**Table 1.** Serological Test Results on the Sera Sampled from Hospitalized Human Patients

Patient groups based on clinical signs	Serological tests			No. of test results
	IgM ELISA	IgG ELISA	Western blot	
Arthritis	Neg^a^	Neg	Neg	3
	Neg	Neg	Nt^b^	11
	Neg	Pos	Neg	5
	Pos^a^	Neg	Pos	10
Erythema migrans-like lesions	Neg	Neg	Neg	15
	Neg	Neg	Nt	7
	Neg	Neg	Pos	7
	Neg	Pos	Neg	4
	Neg	Pos	Pos	5
	Pos	Neg	Neg	2
	Pos	Neg	Pos	11
	Pos	Pos	Neg	2
	Pos	Pos	Pos	7
Neurological signs	Bd^c^	Neg	Nt	1
	Bd	Pos	Pos	1
	Neg	Neg	Nt	24
	Neg	Pos	Neg	7
	Pos	Neg	Pos	1
	Pos	Pos	Pos	1
Pulmonary tuberculosis	Bd	Neg	Nt	3
	Neg	Neg	Nt	61
	Neg	Pos	Neg	9
	Neg	Pos	Nt	1
	Pos	Neg	Pos	1
	Pos	Pos	Pos	4

^a^ Pos and Neg denote positive (>22 relative units per mL [RU/mL]) and negative test (<16 RU/mL) results, respectively.

^b^ Nt denotes nontested.

^c^ Bd denotes borderline test result (16–22 RU/mL).

The diagnosis of LB was considered confirmatory for those patients who tested positive by IgM or IgG ELISA and by WB. LB was confirmed in 8.6%, 34.5%, and 50% of the patients with neurological signs, arthritis, or EM-like lesions, respectively. The laboratory testing was consistent with LB in 6.3% of the patients with pulmonary tuberculosis (Table 1). Thus, 23.6% (48 out of 203) of the patients were serologically positive for LB, including those 7 patients who were WB positive but ELISA negative. The other 41 LB-confirmed patients tested positive by IgM or IgG ELISA and WB (*n* = 29) or by the three assays (*n* = 12). Overall, the duration of clinical signs (including pulmonary tuberculosis) was significantly shorter for the 48 LB-confirmed patients (median = 37 days) compared with the other 45 ELISA-positive but WB-negative patients (median = 90 days) (*p* < 0.05). There was no significant association identified between different combinations of ELISA- and/or WB-positive results and age or gender of the tested patients. The cutaneous LB was confirmed in 30 out of 32 patients (93.8%) who had developed EM, the pathognomonic lesion of LB (Hatchette et al. 2014). Of note, 25.8% of ELISA-positive patients with EM-like lesions tested negative by WB. The skin lesions of the 8 falsely positive patients were diagnosed as morphea (*n* = 6) or psoriasis (*n* = 2). In comparison, substantially higher numbers of falsely positive ELISA test results were observed for the arthritic (33.3%), neurologic (70%), and pulmonary tuberculosis-confirmed (64.3%) patients (Table 1). Together, 29 out of 70 ELISA-positive results (41%) were not confirmed by WB, reiterating the requirement of this serological assay to be a confirmatory step in serology-based LB diagnostic protocols (Eldin et al. 2019).

In summary, this study is the first to provide the data on the seroprevalence of LB in a subset of hospitalized population in Ukraine. The study examined 203 patients and showed that the LB laboratory testing was confirmatory for 8.6%, 34.5%, and 50% of the patients with symptoms compatible with LB. Future studies that would examine different subsets of clinically healthy population across various regions of Ukraine are highly warranted.

## Supplementary Material

Supplemental data

Supplemental data

Supplemental data

Supplemental data

## References

[B1] Eldin C, Raffetin A, Bouiller K, Hansmann Y, et al. Review of European and American guidelines for the diagnosis of Lyme borreliosis. Med Mal Infect 2019; 49:121–132 30528068 10.1016/j.medmal.2018.11.011

[B2] Hatchette TF, Davis I, Johnston BL. Lyme disease: Clinical diagnosis and treatment. Can Commun Dis Rep 2014; 40:194–208 29769842 10.14745/ccdr.v40i11a01 PMC5864449

[B3] Kuehn BM. CDC estimates 300,000 US cases of Lyme disease annually. JAMA 2013; 310:1110 24045727 10.1001/jama.2013.278331

[B4] Littman MP, Gerber B, Goldstein RE, Labato MA, et al. ACVIM consensus update on Lyme borreliosis in dogs and cats. J Vet Intern Med 2018; 32:887–903 29566442 10.1111/jvim.15085 PMC5980284

[B5] Rogovskyy AS, Biatov AP, Davis MA, Liu S, et al. Upsurge of Lyme borreliosis in Ukraine: A 20-year survey. J Travel Med 2020; 27:taaa100 32577753 10.1093/jtm/taaa100

[B6] Sykes RA, Makiello P. An estimate of Lyme borreliosis incidence in Western Europe. J Public Health (Oxford, England) 2017; 39:74–81 10.1093/pubmed/fdw017 26966194

[B7] Vandekerckhove O, De Buck E, Van Wijngaerden E. Lyme disease in Western Europe: An emerging problem? A systematic review. Acta Clin Belg 2019. [Epub ahead of Print]; DOI: 10.1080/17843286.2019.1694293 31739768

