# Supplementary material for: Serological Surveillance of Hospitalized Patients for Lyme Borreliosis in Ukraine
Source: Vector Borne Zoonotic Dis. 2021 Mar 25;21(4):301–3. doi: 10.1089/vbz.2020.2715 (PMC7997712; doi:10.1089/vbz.2020.2715)
Supplement: Supplemental data [file Supp_Table1.docx]

**Supplemental Table S1.** Serological test results on the sera sampled from human patients with arthritis.

| Patient ID | Sex | Age (years) | Diagnosis | Other clinical symptoms | History of tick bites | Duration of arthritis (months) | Anti-*Borrelia* IgM ELISA | Anti-*Borrelia* IgG ELISA | Western blot |
| --- | --- | --- | --- | --- | --- | --- | --- | --- | --- |
| Msd 1 | M^a^ | 52 | Arthritis | None | Yes | 2 | Pos^b^ | Neg^b^ | Pos |
| Msd 2 | F^a^ | 31 | Arthritis | Fever, myalgia | Yes | 4 | Pos | Neg | Pos |
| Msd 3 | M | 29 | Arthritis | None | Yes | 2 | Pos | Neg | Pos |
| Msd 4 | F | 54 | Arthritis | Myalgia | No | 2.5 | Neg | Neg | Nt^c^ |
| Msd 5 | F | 37 | Arthritis | None | Yes | 3 | Neg | Neg | Nt |
| Msd 6 | M | 24 | Arthritis | None | Yes | 5 | Neg | Pos | Neg |
| Msd 7 | M | 48 | Arthritis | None | Yes | 4 | Neg | Neg | Nt |
| Msd 8 | F | 45 | Arthritis | Fever, headache | No | 1 | Pos | Neg | Pos |
| Msd 9 | F | 52 | Arthritis | None | No | 5 | Neg | Neg | Nt |
| Msd 10 | F | 29 | Arthritis | Headache | Yes | 1.5 | Pos | Neg | Pos |
| Msd 11 | F | 43 | Arthritis | Fever | No | 2 | Pos | Neg | Pos |
| Msd 12 | F | 59 | Arthritis | Myalgia | Yes | 2.5 | Pos | Neg | Pos |
| Msd 13 | M | 41 | Arthritis | None | Yes | 5 | Neg | Pos | Neg |
| Msd 14 | M | 25 | Arthritis | Headache | No | 3 | Pos | Neg | Pos |
| Msd 15 | M | 62 | Arthritis | Myalgia | No | 1 | Pos | Neg | Pos |
| Msd 16 | M | 37 | Arthritis | Myalgia | Yes | 3.5 | Pos | Neg | Pos |
| Msd 17 | F | 43 | Arthritis | None | No | 3 | Neg | Neg | Nt |
| Msd 18 | F | 51 | Arthritis | None | Yes | 2 | Neg | Neg | Nt |
| Msd 19 | F | 44 | Arthritis | None | Yes | 3 | Neg | Neg | Nt |
| Msd 20 | M | 27 | Arthritis | None | Yes | 6 | Neg | Neg | Nt |
| Msd 21 | F | 34 | Arthritis | Myalgia | No | 6 | Neg | Pos | Neg |
| Msd 22 | F | 38 | Arthritis | None | Yes | 5 | Neg | Neg | Nt |
| Msd 23 | F | 59 | Arthritis | None | Yes | 2.5 | Neg | Neg | Nt |
| Msd 24 | M | 70 | Arthritis | None | Yes | 5 | Neg | Neg | Nt |
| Msd 25 | F | 58 | Arthritis | Myalgia | Yes | 4 | Neg | Pos | Neg |
| Msd 26 | M | 43 | Arthritis | Myalgia | No | 4 | Neg | Pos | Neg |
| Msd 27 | M | 49 | Arthritis | Fever | Yes | 3 | Neg | Neg | Neg |
| Msd 28 | F | 30 | Arthritis | Fever | Yes | 3 | Neg | Neg | Neg |
| Msd 29 | F | 59 | Arthritis | Fever | Yes | 4 | Neg | Neg | Neg |

^a^M and F denote male and female, respectively.

^b^Pos and Neg denote positive and negative test results, respectively.

^c^Nt denotes nontested.
