# Supplementary material for: Serological Surveillance of Hospitalized Patients for Lyme Borreliosis in Ukraine
Source: Vector Borne Zoonotic Dis. 2021 Mar 25;21(4):301–3. doi: 10.1089/vbz.2020.2715 (PMC7997712; doi:10.1089/vbz.2020.2715)
Supplement: Supplemental data [file Supp_Table4.docx]

**Supplemental Table S4.** Serological test results on the sera sampled from human patients with respiratory symptoms.

| Patient ID | Sex | Age (years) | Diagnosis | Other clinical symptoms | History of tick bites | Duration of respiratory symptoms  (months) | Anti-*Borrelia* IgM ELISA | Anti-*Borrelia* IgG ELISA | Western blot |
| --- | --- | --- | --- | --- | --- | --- | --- | --- | --- |
| Rsd 1 | F^a^ | 30 | DPT^b^ | Fever, myalgia, joint pain | Yes | 1 | Pos^c^ | Pos | Pos |
| Rsd 2 | F | 52 | IPT^b^ | Fever, headache | No | 2.5 | Bd^d^ | Neg | Nt^e^ |
| Rsd 3 | F | 50 | EPT^b^ | Headache | Yes | 3 | Neg^c^ | Neg | Nt |
| Rsd 4 | M^a^ | 22 | DPT | Myalgia | No | 1 | Neg | Neg | Nt |
| Rsd 5 | F | 34 | IPT | Fever | No | 3 | Neg | Neg | Nt |
| Rsd 6 | F | 61 | DPT | Fever | Yes | 2 | Neg | Neg | Nt |
| Rsd 7 | F | 50 | DPT | Fever, myalgia | Yes | 6 | Neg | Neg | Nt |
| Rsd 8 | M | 19 | DPT | None | Yes | 1 | Neg | Neg | Nt |
| Rsd 9 | M | 30 | IPT | Headache | Yes | 1.5 | Pos | Neg | Pos |
| Rsd 10 | M | 45 | EPT | Fever, headache | Yes | 3.5 | Neg | Pos | Neg |
| Rsd 11 | F | 23 | IPT | None | Yes | 1 | Neg | Neg | Nt |
| Rsd 12 | M | 30 | DPT | Headache | Yes | 2 | Pos | Pos | Pos |
| Rsd 13 | F | 44 | DPT | Headache | Yes | 2 | Neg | Neg | Nt |
| Rsd 14 | F | 55 | IPT | None | Yes | 1 | Neg | Neg | Nt |
| Rsd 15 | M | 63 | IPT | Myalgia | Yes | 2 | Neg | Neg | Nt |
| Rsd 16 | M | 33 | EPT | Fever, headache | Yes | 2 | Neg | Neg | Nt |
| Rsd 17 | F | 56 | IPT | None | Yes | 2 | Neg | Neg | Nt |
| Rsd 18 | F | 51 | IPT | Myalgia | No | 1 | Neg | Neg | Nt |
| Rsd 19 | M | 23 | DPT | Fever, headache | No | 2 | Neg | Neg | Nt |
| Rsd 20 | M | 39 | DPT | Fever, headache | Yes | 3 | Bd | Neg | Nt |
| Rsd 21 | M | 59 | IPT | None | Yes | 5 | Neg | Neg | Nt |
| Rsd 22 | F | 19 | EPT | Headache | No | 4 | Neg | Pos | Neg |
| Rsd 23 | F | 45 | IPT | Myalgia | No | 2.5 | Neg | Neg | Nt |
| Rsd 24 | F | 65 | IPT | Fever, joint pain | No | 2 | Neg | Pos | Neg |
| Rsd 25 | M | 49 | DPT | None | Yes | 1.5 | Neg | Neg | Nt |
| Rsd 26 | M | 35 | IPT | Headache, joint pain | Yes | 5.5 | Neg | Neg | Nt |
| Rsd 27 | M | 70 | DPT | None | Yes | 2 | Neg | Neg | Nt |
| Rsd 28 | F | 60 | DPT | Myalgia | Yes | 1 | Neg | Pos | Neg |
| Rsd 29 | F | 27 | IPT | Fever, headache | Yes | 5 | Neg | Neg | Nt |
| Rsd 30 | M | 21 | EPT | Fever | Yes | 2 | Neg | Neg | Nt |
| Rsd 31 | F | 41 | DPT | Headache | No | 4.5 | Neg | Neg | Nt |
| Rsd 32 | F | 32 | DPT | Headache | Yes | 3.5 | Pos | Pos | Pos |
| Rsd 33 | F | 33 | DPT | None | Yes | 1 | Neg | Neg | Nt |
| Rsd 34 | F | 52 | DPT | None | Yes | 1 | Neg | Neg | Nt |
| Rsd 35 | M | 55 | IPT | Fever, myalgia | No | 2 | Neg | Neg | Nt |
| Rsd 36 | M | 42 | IPT | None | Yes | 0.5 | Neg | Neg | Nt |
| Rsd 37 | F | 19 | DPT | Myalgia | Yes | 1.5 | Neg | Neg | Nt |
| Rsd 38 | M | 60 | DPT | Fever, myalgia | Yes | 1 | Neg | Neg | Nt |
| Rsd 39 | F | 63 | DPT | Headache | No | 0.5 | Neg | Pos | Neg |
| Rsd 40 | F | 46 | IPT | Fever | Yes | 3.5 | Neg | Pos | Neg |
| Rsd 41 | F | 35 | DPT | Fever, headache | Yes | 2 | Neg | Pos | Neg |
| Rsd 42 | M | 59 | IPT | None | Yes | 1.5 | Neg | Neg | Nt |
| Rsd 43 | M | 49 | DPT | Myalgia | No | 0.5 | Neg | Neg | Nt |
| Rsd 44 | F | 28 | DPT | Myalgia | Yes | 2.5 | Bd | Neg | Nt |
| Rsd 45 | F | 57 | DPT | None | Yes | 1 | Neg | Neg | Nt |
| Rsd 46 | F | 35 | IPT | None | Yes | 3 | Neg | Neg | Nt |
| Rsd 47 | M | 65 | IPT | Fever | Yes | 1 | Neg | Neg | Nt |
| Rsd 48 | M | 61 | DPT | None | Yes | 2 | Neg | Neg | Nt |
| Rsd 49 | F | 58 | DPT | Fever | No | 1 | Neg | Neg | Nt |
| Rsd 50 | F | 36 | DPT | Fever | No | 1 | Neg | Neg | Nt |
| Rsd 51 | M | 47 | IPT | Fever, headache | Yes | 2 | Neg | Neg | Nt |
| Rsd 52 | F | 40 | DPT | None | Yes | 1 | Neg | Neg | Nt |
| Rsd 53 | F | 21 | IPT | Myalgia | Yes | 2 | Neg | Neg | Nt |
| Rsd 54 | F | 29 | IPT | None | Yes | 1 | Neg | Neg | Nt |
| Rsd 55 | M | 59 | IPT | Fever, headache | Yes | 1 | Neg | Neg | Nt |
| Rsd 56 | M | 30 | DPT | Fever | Yes | 2 | Neg | Pos | Neg |
| Rsd 57 | F | 19 | DPT | None | Yes | 1 | Neg | Neg | Nt |
| Rsd 58 | F | 60 | IPT | None | Yes | 9 | Neg | Neg | Nt |
| Rsd 59 | F | 37 | DPT | None | Yes | 2 | Neg | Neg | Nt |
| Rsd 60 | M | 22 | IPT | Myalgia | Yes | 2.5 | Neg | Neg | Nt |
| Rsd 61 | F | 41 | DPT | Fever | Yes | 1 | Neg | Neg | Nt |
| Rsd 62 | M | 67 | IPT | None | Yes | 1 | Neg | Neg | Nt |
| Rsd 63 | F | 50 | IPT | None | Yes | 4 | Neg | Neg | Nt |
| Rsd 64 | F | 51 | DPT | None | Yes | 3 | Neg | Neg | Nt |
| Rsd 65 | F | 30 | DPT | Headache | Yes | 6 | Neg | Pos | Neg |
| Rsd 66 | M | 29 | IPT | Fever | Yes | 2 | Neg | Neg | Nt |
| Rsd 67 | M | 51 | DPT | Fever, myalgia | Yes | 2.5 | Neg | Pos | Nt |
| Rsd 68 | F | 44 | IPT | None | Yes | 3.5 | Neg | Neg | Nt |
| Rsd 69 | F | 33 | IPT | Fever | Yes | 1.5 | Pos | Pos | Pos |
| Rsd 70 | F | 62 | DPT | Headache | Yes | 5 | Neg | Neg | Nt |
| Rsd 71 | M | 55 | DPT | None | Yes | 1 | Neg | Neg | Nt |
| Rsd 72 | M | 37 | IPT | None | Yes | 3 | Neg | Neg | Nt |
| Rsd 73 | F | 20 | IPT | Fever, myalgia | Yes | 1 | Neg | Neg | Nt |
| Rsd 74 | F | 39 | DPT | None | Yes | 1 | Neg | Neg | Nt |
| Rsd 75 | M | 40 | DPT | Fever | Yes | 3 | Neg | Neg | Nt |
| Rsd 76 | F | 65 | IPT | Myalgia | Yes | 3 | Neg | Neg | Nt |
| Rsd 77 | F | 61 | IPT | Headache | Yes | 1 | Neg | Neg | Nt |
| Rsd 78 | M | 32 | DPT | None | Yes | 2 | Neg | Neg | Nt |
| Rsd 79 | F | 49 | DPT | None | Yes | 3.5 | Neg | Neg | Nt |

^a^F and M denote male and female, respectively.

^b^DTP, IPT, and EPT denote disseminated pulmonary tuberculosis, infiltrative pulmonary tuberculosis, and extra-pulmonary tuberculosis, respectively.

^c^Pos and Neg denote positive and negative test results, respectively.

^d^Bd denotes borderline test result (16-22 relative units per ml [RU/ml]).

^e^Nt denotes nontested.
