# Supplementary material for: Serological Surveillance of Hospitalized Patients for Lyme Borreliosis in Ukraine
Source: Vector Borne Zoonotic Dis. 2021 Mar 25;21(4):301–3. doi: 10.1089/vbz.2020.2715 (PMC7997712; doi:10.1089/vbz.2020.2715)
Supplement: Supplemental data [file Supp_Table2.docx]

**Supplemental Table S2.** Serological test results on the sera sampled from human patients with skin disorders.

| Patient ID | Sex | Age (years) | Diagnosis | Other clinical symptoms | History of tick bites | Duration of skin disease | Anti-*Borrelia* IgM ELISA | Anti-*Borrelia* IgG ELISA | Western blot |
| --- | --- | --- | --- | --- | --- | --- | --- | --- | --- |
| Sd 1 | F^a^ | 46 | Erythrema migrans | None | Yes | 37 days | Pos^b^ | Neg^b^ | Pos |
| Sd 2 | M^a^ | 38 | Erythrema migrans | None | No | 40 days | Neg | Pos | Pos |
| Sd 3 | M | 34 | Erythrema migrans | None | Yes | 14 days | Pos | Pos | Pos |
| Sd 4 | F | 27 | Morphea | None | Yes | 2 years | Pos | Neg | Neg |
| Sd 5 | F | 56 | Erythrema migrans | None | No | 65 days | Neg | Pos | Pos |
| Sd 6 | F | 50 | Morphea | None | Yes | 4 years | Pos | Pos | Neg |
| Sd 7 | F | 36 | Erythrema migrans | None | No | 42 days | Pos | Neg | Pos |
| Sd 8 | M | 42 | Erythrema migrans | None | Yes | 8 days | Pos | Neg | Pos |
| Sd 9 | F | 42 | Erythrema migrans | None | Yes | 34 days | Pos | Pos | Pos |
| Sd 10 | M | 18 | Psoriasis | None | Yes | 1 year | Pos | Pos | Neg |
| Sd 11 | F | 61 | Morphea | None | No | 6 (month) | Neg | Neg | Nt^c^ |
| Sd 12 | F | 54 | Morphea | None | Yes | 8 (month) | Neg | Pos | Neg |
| Sd 13 | F | 32 | Psoriasis | None | No | 1 (year) | Pos | Neg | Neg |
| Sd 14 | F | 28 | Erythrema migrans | None | Yes | 75 (days) | Pos | Pos | Pos |
| Sd 15 | M | 22 | Erythrema migrans | Fever, joint pain | Yes | Unknown | Neg | Neg | Pos |
| Sd 16 | M | 63 | Erythrema migrans | None | No | Unknown | Pos | Neg | Pos |
| Sd 17 | F | 59 | Morphea | None | Yes | 3 (years) | Neg | Neg | Nt |
| Sd 18 | F | 33 | Erythrema migrans | None | No | 27 (days) | Pos | Pos | Pos |
| Sd 19 | F | 34 | Psoriasis | None | Yes | 1 (year) | Neg | Neg | Nt |
| Sd 20 | M | 52 | Erythrema migrans | None | Yes | 4 (days) | Pos | Neg | Pos |
| Sd 21 | M | 26 | Lichen planus | None | Yes | 75 (days) | Neg | Neg | Nt |
| Sd 22 | F | 30 | Erythrema migrans | None | Yes | 19 (days) | Pos | Neg | Pos |
| Sd 23 | F | 41 | Lichen planus | None | No | 95 (days) | Neg | Neg | Nt |
| Sd 24 | F | 37 | Erythrema migrans | None | No | 27 (days) | Pos | Pos | Pos |
| Sd 25 | F | 48 | Urticaria | None | No | 229 (days) | Neg | Neg | Nt |
| Sd 26 | M | 42 | Erythrema migrans | Fever, joint pain | Yes | Unknown | Neg | Neg | Nt |
| Sd 27 | M | 57 | Urticaria | None | Yes | 27 (days) | Neg | Neg | Neg |
| Sd 28 | F | 55 | Urticaria | None | Yes | 35 (days) | Neg | Neg | Neg |
| Sd 26 | M | 42 | Erythrema migrans | Fever, joint pain | Yes | Unknown | Neg | Neg | Pos |
| Sd 32 | M | 32 | Erythrema migrans | Fatigue | Yes | 5 (days) | Neg | Neg | Pos |
| Sd 33 | M | 29 | Erythrema migrans | None | Yes | 19 (days) | Pos | Neg | Pos |
| Sd 34 | F | 73 | Erythrema migrans | Fatigue, joint pain | Yes | 30 (days) | Neg | Neg | Pos |
| Sd 35 | F | 59 | Morphea | None | Yes | 1 (year) | Neg | Pos | Neg |
| Sd 36 | F | 55 | Erythrema migrans | None | No | 37 (days) | Pos | Neg | Pos |
| Sd 38 | F | 40 | Erythrema migrans | Fever, fatigue | Yes | 30 (days) | Neg | Neg | Neg |
| Sd 39 | F | 42 | Erythrema migrans | Fever, joint pain | Yes | 253 (days) | Neg | Neg | Pos |
| Sd 40 | F | 51 | Erythrema migrans | None | Yes | 81 (days) | Pos | Pos | Pos |
| Sd 41 | F | 35 | Erythrema migrans | None | No | 30 (days) | Pos | Neg | Pos |
| Sd 43 | F | 48 | Erythrema migrans | Fatigue, joint pain | Yes | 50 (days) | Neg | Neg | Pos |
| Sd 44 | F | 64 | Erythrema migrans | None | Yes | 90 (days) | Neg | Pos | Pos |
| Sd 45 | M | 62 | Erythrema migrans | None | Yes | 15 (days) | Pos | Pos | Pos |
| Sd 48 | F | 40 | Erythrema migrans | Fever, joint pain | Yes | 28 (days) | Neg | Neg | Pos |
| Sd 49 | F | 47 | Erythrema migrans | None | Yes | 57 (days) | Pos | Neg | Pos |
| Sd 50 | F | 68 | Erythrema migrans | None | Yes | 15 (days) | Neg | Pos | Pos |
| Sd 51 | F | 32 | Urticaria | None | Yes | 58 (days) | Neg | Neg | Neg |
| Sd 52 | M | 39 | Erythrema migrans | None | Yes | 35 (days) | Neg | Pos | Pos |
| Sd 53 | F | 22 | Erythrema migrans | None | No | Unknown | Pos | Neg | Pos |
| Sd 54 | F | 32 | Lichen planus | Fever, joint pain | Yes | 90 (days) | Neg | Neg | Neg |
| Sd 55 | M | 47 | Urticaria | Fever, joint pain | Yes | 19 (days) | Neg | Neg | Neg |
| Sd 56 | M | 22 | Urticaria | Fever, joint pain | Yes | 28 (days) | Neg | Neg | Neg |
| Sd 59 | M | 48 | Morphea | None | Yes | 2.5 (years) | Neg | Pos | Neg |
| Sd 60 | M | 29 | Morphea | None | Yes | 3 (years) | Neg | Pos | Neg |
| Sd 61 | M | 33 | Psoriasis | None | Yes | 35 (days) | Neg | Neg | Neg |
| Sd 68 | M | 54 | Urticaria | None | Yes | 48 (days) | Neg | Neg | Neg |
| Sd 74 | F | 43 | Lichen planus | Fatigue, joint pain | Yes | 8 (month) | Neg | Neg | Neg |
| Sd 75 | F | 38 | Psoriasis | None | Yes | 1 (year) | Neg | Neg | Neg |
| Sd 76 | M | 23 | Psoriasis | None | Yes | 1 (year) | Neg | Neg | Neg |
| Sd 77 | M | 67 | Morphea | Fatigue, joint pain | Yes | 3.6 (year) | Neg | Neg | Neg |
| Sd 78 | F | 50 | Morphea | Fatigue, joint pain | Yes | 1 (year) | Neg | Neg | Neg |
| Sd 79 | F | 39 | Urticaria | Fatigue, joint pain | Yes | 45 (days) | Neg | Neg | Neg |

^a^M and F denote male and female, respectively.

^b^Pos and Neg denote positive and negative test results, respectively.

^c^Nt denotes nontested.
