# Supplementary material for: Serological Surveillance of Hospitalized Patients for Lyme Borreliosis in Ukraine
Source: Vector Borne Zoonotic Dis. 2021 Mar 25;21(4):301–3. doi: 10.1089/vbz.2020.2715 (PMC7997712; doi:10.1089/vbz.2020.2715)
Supplement: Supplemental data [file Supp_Table3.docx]

**Supplemental Table S3.** Serological test results on the sera sampled from human patients with neurological signs.

| Patient ID | Sex | Age (years) | Diagnosis | Other clinical symptoms | History of tick bites | Duration of neurological symptoms (days) | Anti-*Borrelia* IgM ELISA | Anti-*Borrelia* IgG ELISA | Western blot |
| --- | --- | --- | --- | --- | --- | --- | --- | --- | --- |
| Nsd 1 | M^a^ | 22 | Peripheral neuritis | None | Yes | 7 | Neg^b^ | Neg | Nt^c^ |
| Nsd 2 | F^a^ | 50 | Cranial neuritis | Headache | Yes | 9 | Pos^b^ | Neg | Pos |
| Nsd 3 | F | 33 | Peripheral neuritis | None | Yes | 9 | Neg | Neg | Nt |
| Nsd 4 | F | 49 | Neuritis of the facial nerve | Myalgia | Yes | 8 | Neg | Neg | Nt |
| Nsd 5 | M | 30 | Peripheral neuritis | Fever, myalgia | Yes | 7 | Pos | Pos | Pos |
| Nsd 6 | M | 28 | Cranial neuritis | Myalgia | No | 7 | Neg | Neg | Nt |
| Nsd 7 | M | 37 | Painful meningoradiculitis | Fever, myalgia | No | 6 | Neg | Neg | Nt |
| Nsd 8 | F | 19 | Cranial neuritis | None | Yes | 7 | Neg | Neg | Nt |
| Nsd 9 | F | 52 | Cranial neuritis | Headache | Yes | 9 | Neg | Neg | Nt |
| Nsd 10 | F | 46 | Neuritis of the facial nerve | Fever, headache, | Yes | 7 | Bd^d^ | Pos | Pos |
| Nsd 11 | M | 34 | Painful meningoradiculitis | None | No | 8 | Neg | Neg | Nt |
| Nsd 12 | F | 41 | Multiple sclerosis | None | Yes | 9 | Neg | Neg | Nt |
| Nsd 13 | M | 49 | Peripheral neuritis | Fever | Yes | 6 | Neg | Neg | Nt |
| Nsd 14 | F | 30 | Cranial neuritis | Fever | No | 6 | Neg | Neg | Nt |
| Nsd 15 | F | 27 | Neuritis of the facial nerve | Fever, myalgia | Yes | 7 | Neg | Pos | Neg |
| Nsd 16 | F | 30 | Multiple sclerosis | Fever | Yes | 7 | Neg | Pos | Neg |
| Nsd 17 | M | 52 | Peripheral neuritis | None | Yes | 8 | Neg | Neg | Nt |
| Nsd 18 | F | 40 | Cranial neuritis | None | Yes | 8 | Neg | Neg | Nt |
| Nsd 19 | F | 60 | Peripheral neuritis | None | Yes | 6 | Neg | Neg | Nt |
| Nsd 20 | F | 32 | Neuritis of the facial nerve | None | Yes | 6 | Neg | Neg | Nt |
| Nsd 21 | M | 42 | Painful meningoradiculitis | Myalgia | Yes | 7 | Neg | Neg | Nt |
| Nsd 22 | M | 48 | Cranial neuritis | Myalgia | No | 8 | Bd | Neg | Nt |
| Nsd 23 | F | 19 | Peripheral neuritis | Fever, joint pain, myalgia | Yes | 6 | Neg | Pos | Neg |
| Nsd 24 | F | 26 | Painful meningoradiculitis | None | Yes | 6 | Neg | Neg | Nt |
| Nsd 25 | F | 38 | Multiple sclerosis | None | Yes | 9 | Neg | Neg | Nt |
| Nsd 26 | M | 30 | Neuritis of the facial nerve | Fever, myalgia | No | 8 | Neg | Neg | Nt |
| Nsd 27 | F | 58 | Neuritis of the facial nerve | Fever, myalgia | Yes | 7 | Neg | Neg | Nt |
| Nsd 28 | M | 47 | Peripheral neuritis | Joint pain | Yes | 6 | Neg | Pos | Neg |
| Nsd 29 | F | 21 | Peripheral neuritis | Joint pain | Yes | 8 | Neg | Pos | Neg |
| Nsd 30 | M | 63 | Multiple sclerosis | None | Yes | 7 | Neg | Neg | Nt |
| Nsd 31 | F | 32 | Peripheral neuritis | Headache | Yes | 8 | Neg | Neg | Nt |
| Nsd 32 | F | 25 | Neuritis of the facial nerve | None | Yes | 8 | Neg | Neg | Nt |
| Nsd 33 | F | 54 | Neuritis of the facial nerve | None | Yes | 7 | Neg | Neg | Nt |
| Nsd 34 | M | 50 | Multiple sclerosis | None | Yes | 8 | Neg | Pos | Neg |
| Nsd 35 | M | 19 | Neuritis of the facial nerve | Fever, joint pain | No | 8 | Neg | Pos | Neg |

^a^M and F denote male and female, respectively.

^b^Pos and Neg denote positive and negative test results, respectively.

^c^Nt denotes nontested.

^d^Bd denotes borderline test result (16-22 relative units per ml [RU/ml]).
